# Effect of Thermal Processing on Color, Phenolic Compounds, and Antioxidant Activity of Faba Bean (*Vicia faba* L.) Leaves and Seeds

**DOI:** 10.3390/antiox10081207

**Published:** 2021-07-28

**Authors:** Shu-Cheng Duan, Soon-Jae Kwon, Seok-Hyun Eom

**Affiliations:** 1 Department of Horticultural Biotechnology, College of Life Sciences, Kyung Hee University, Yongin 17104, Korea; dsc97@khu.ac.kr; 2 Advanced Radiation Technology Institute, Korea Atomic Energy Research Institute, Jeongeup 56212, Korea; soonjaekwon@kaeri.re.kr

**Keywords:** faba bean, seeds, leaves, thermal processing, L-dopa, antioxidant

## Abstract

The leaves and seeds of the faba bean are good sources of L-3,4-dihydroxyphenylalanin (L-dopa), and are usually eaten with thermal cooking methods. However, little information is available on the effect of thermal treatments on their nutritional value. We compared the changes in color, contents of L-dopa, vitamin C (Vc), total phenolics (TP), total flavonoids (TF) and antioxidant activity after dry heating or steaming faba bean leaves and seeds. The young leaves provided higher values of all the estimate factors, regardless of the thermal treatment. Steaming significantly degraded nutritional values of the leaves, but less changed in seeds, whereas dry heat maintained these attributes. The contents of L-dopa, Vc, TP and TF were shown to have strongly positive correlations with antioxidant activity in the leaves, whereas only L-dopa content was positively correlated with antioxidant activity of the seeds. Faba leaves contained relatively high L-dopa which possessed strong antioxidant activity compared to the Vc. As L-dopa is an important contributor to the antioxidant activity of faba leaves and seeds, consuming L-dopa from leaves may provide beneficial effects not only regarding Parkinson’s Disease.

## 1. Introduction

Faba beans (*Vicia faba* L., Fabaceae family) play an important role among the legumes of the world. They are cultivated in many countries because of the valuable nutrients and special flavor of the seeds [1]. During the cultivation period of faba beans, ranging from 80 to 120 days [2,3], the necessary green pruning will generate a large number of by-products, such as leaves, stems and flowers [3,4]. However, these types of by-product are mainly disposed of as waste because of the traditional agriculture practice of focusing on harvesting the multi-functional mature seeds. Nevertheless, faba leaves are commonly consumed in many countries as a vegetable similar to spinach using several domestic cooking methods for consumption in salads, as a boiled vegetable or baked in omelets [4]. Faba leaves have also been used in traditional Chinese folk medicine for hemostasis and detoxication [5].

Faba seeds have been studied extensively and widely used in the food and pharmaceutical industries due to its properties on enhancement of nutrition or reduction the complications and progression of certain diseases [6,7,8]. Faba leaves have also gained attention as an unconventional vegetable, with several studies providing significant results on characterizing their phytochemicals and proximate nutritional values [4,9,10,11]. Faba leaves have also been considered to have development potential as a food or medicinal material, similar to faba seeds. This is mainly due to the presence of L-dopa, a type of phenolic acid in faba leaves considered since the last century as an important drug for treating the effects of Parkinson’s disease (PD) [12]. L-dopa has also been reported to improve color vision, prevent hypertension and reduce the risk of renal failure [13,14]. Faba seeds have long been recognized as a good edible natural source of L-dopa, while in recent studies, leaves have been suggested to possess more than ten times the content of L-dopa than seeds [11,15]. The relatively strong antioxidant activity of faba leaves (supply about 11.5 mmol of trolox equivalent 100 g^−1^ fresh weight) compared with commercial vegetables (lower than 6 mmol of trolox equivalent 100 g^−1^ fresh weight) such as broccoli, asparagus, spinach, cabbage and green peas have also attracted much scientific attention [4]. The polyphenols (vitamins, phenolics and flavonoids) are usually regarded as important contributors to the antioxidant potential and chemopreventive properties of food materials [16,17,18,19]. However, compared with the information available on faba seeds [1,20], few studies have evaluated the polyphenol contents of the faba leaf and its contribution to antioxidant activity.

Application of thermal processing in food matrix is one of the common methods for several purposes, like prolong of food shelf-life and improvement of tastes, texture, color, nutritional value, and so on. [16,17,21]. Many studies have reported on the changes in different properties of various phytochemicals in faba seeds after thermal treatment, such as the increase of phytic acid and the decrease of tannins contents after microwave, ordinary cooking, or autoclave treatments compared with raw seeds [22,23]. However, compared with faba seeds, the effect of thermal processing on the phytochemical content and antioxidant activity of faba leaves at different levels of maturity has not been well-studied.

Therefore, the present study aimed to evaluate the nutritional value of faba leaves by comparing the effect of dry and wet (steaming) thermal processing on their color, contents of bioactive compounds (L-dopa, vitamin C, total phenolics, total flavonoids) and antioxidant activity (DPPH and ABTS radical-scavenging abilities) in faba seeds and faba leaves at different levels of maturity. The relationship between the content of bioactive compounds and the antioxidant activity of each material will also be determined by correlation analysis. The results of the present study will directly provide much-needed information on the use of faba leaves in the daily diet and indirectly contribute to improving the breeding of faba cultivars for specific use of the leaves as a vegetable.

## 2. Materials and Methods

### 2.1. Chemicals and Materials

Analytical standards of L-dopa and vitamin C were purchased from Sigma-Aldrich Co. (St. Louis, MO, USA) and Daejung Chemicals & Metals Co (Siheung, Korea), respectively. Other chemicals and solvents used were of analytical grade.

Faba bean seeds (Accession number: PI 577722) harvested in 2020 were provided by the Advanced Radiation Technology Institute (Jeongeup, Korea). For the faba leaf materials, we planted seeds in the greenhouse of Kyung Hee University (37°14′36.0″ N 127°04′52.6″ E, Yongin, Korea) in December, 2020, followed a previous reported method [11]. In brief, the seeds were soaked in distilled water for 24 h, then washed off the surface mucus. The seeds that are healthy and intact were selected, and planted in a pot filled with horticultural soil (Baroker, Seoulbio Co., Eumseong, Korea). The plants relied on natural light and the temperature of the greenhouse was set at 20 ± 5 °C. According to the plant growth condition, watering the plants 3–5 times per week with the purpose to maintaining adequate water content in soil. Leaf materials of different maturity were collected in March, 2021. Young (on the apex) and old (on the base) leaves were gathered separately, washed with distilled water then wiped with tissue paper. The intact and undamaged leaves and seeds were selected for further processing.

### 2.2. Processing

Twenty gram of fresh leaves (young and old, respectively)/ten of seeds were collected and lyophilized at −67 °C using a freeze dryer (IlshinBioBase, Dongducheon, Korea) as control group. All of the totally dried samples (the below treated sample also) were ground using a mortar and a pestle, and sieved with a 100-mesh for further analysis.

#### 2.2.1. Dry Heat Treatment

Batches of fresh leaves (same amount as above) and seeds (10) were heated to 100 °C in a convective dry heat machine (Koencon Co., Ltd., Hanam, Korea) for 15, 30, 45 or 60 min. After treatment, each sample was dried at 30 °C to a constant weight.

#### 2.2.2. Wet Heat Treatment (Steaming)

One liter of distilled water was placed in a pot with a tray and a lid. Batches of fresh leaves (same amount as above) and seeds (10) were placed on the tray when the water was boiling then covered by the lid immediately. Under atmospheric conditions, the steaming time was set at 15, 30, 45 or 60 min. The treated samples were then cooled by an ice pack and the surface water was removed using gauze. The samples were then dried at 30 °C to a constant weight.

### 2.3. Measurement of Color Variations

The color of each dried sample was measured by a color analyzer (RGB system) (Lutron Electronics, Inc., Coopersburg, PA, USA). The values of L* (lightness/darkness), a* (redness/greenness) and b* (yellowness/blueness) were converted from RGB values using OpenRGB software (version 2.30.10125, Logicol S.r.l., Trieste, Italy).

### 2.4. Sample Extraction

#### 2.4.1. For L-dopa, Total Phenolics, Total Flavonoids and Antioxidant Activity Measurements

The sample extraction method is described by Neugart et al. [10] and Polanowska et al. [24] with some modifications. Samples of dried leaves (20 mg) or processed seeds (50 mg) were immersed in 1 mL of 50% aqueous methanol for 60 min with sonication at below 40 °C. The samples were then centrifuged at 14,240× *g* for 15 min and the supernatants collected for further analysis. 

#### 2.4.2. For Vitamin C Content Determination

The extraction method was as described by Nishiyama et al. [25] with some modifications. Twenty milligram of leaf powder or 100 mg of seed powder were combined with 1 mL of ice-cold 8% acetic acid in a brown tube. The mixture was then vortexed for 60 s. The suspension was centrifuged at 14,240× *g* at 4 °C for 10 min. The supernatant after filtration with 0.45-μm membrane filter was analyzed by high-performance liquid chromatograph (HPLC) as shown in Section 2.6.

### 2.5. HPLC Analysis of L-dopa Content 

One milliliter of sample solution (2 mg/mL for leaves; 10 mg/mL for seeds) after filtration through a 0.45-μm membrane filter (Futecs Co., Ltd., Daejeon, Korea) was analyzed by gradient elution on an octadecylsilane column (Prontosil 120-5-C18-SH 5.0 μm (250 × 4.6 mm), Bischoff, Leonberg, Germany) with a Waters 2695 Alliance HPLC (Waters Inc., Milford, MA, USA). An injection volume of 5 μL and a flow rate of 0.8 mL/min were used. The mobile phases were solvent A (0.3% formic acid in water) and solvent B (0.3% formic acid in acetonitrile) with a linear gradient as follows: 0–9 min, 2% B; 9–10 min, 2–80% B; 10–14 min, 80% B; 14–16 min, 80–2% B; and 16–20 min, 2% B. The peak of L-dopa was detected at 280 nm by a Waters 996 photodiode array detector (Waters Inc., Milford, MA, USA).

### 2.6. HPLC Analysis of Vc Content

The sample was analyzed by same HPLC systems as in Section 2.5. The separation was carried out on a Hypersil GOLD C18 column (5 μm, 150 × 4.6 mm, Thermo Fisher Scientific, Waltham, MA, USA). The flow rate was 1 mL/min with an injection volume of 10 μL. The mobile phase consisted of solvent A (water with 2% formic acid) and solvent B (acetonitrile with 2% formic acid) applied with a linear gradient as follows: 0–1 min, 0–5% B; 1–5 min, 5–9.4% B; 5–6 min, 9.4–0% B; 6–10 min, 0% B. The peak of vitamin C was monitored at 254 nm.

### 2.7. Measurement of Total Phenolics Content (TPC) and Total Flavonoids Content (TFC)

The total content of phenolic compounds was determined using the Folin-Ciocalteu method as described by Lim et al. [26] with some modifications. In brief, 50 μL of the sample solution (2 mg/mL for leaves; 10 mg/mL for seeds) were added to a 1.5-mL tube containing 650 μL of distilled water. Then 50 μL of Folin-Ciocalteu phenol reagent (Sigma-Aldrich Co.) were added and immediately mixed by vortexing. After 6 min, 500 μL of 7% of Na_2_CO_3_ were added to the tube, and incubated for 90 min at ambient temperature. The absorbance was measured at 750 nm using a spectrophotometer (S-4100; Scinco Co., Seoul, Korea). The total phenolic compounds content was defined in mg of garlic acid equivalent (GAE)/g dry weight (d.w.).

The total flavonoids content was determined by a previously-described colorimetric method [26] with some modifications. Briefly, 100 μL of the sample solution (2 mg/mL for leaves; 50 mg/mL for seeds) were mixed with 640 μL of distilled water and 30 μL of 5% NaNO_2_. After 5 min, 30 μL of 10% AlCl_3_ were added then after mixing for 1 min, 200 μL of 1 M NaOH were also added. The absorbance was measured immediately by a spectrophotometer at 510 nm. The total flavonoids content was defined in mg of catechin equivalent (CE)/g d.w.

### 2.8. Measurement of Antioxidant Activity

The antioxidant activity of samples (leaves and seeds) and standard compounds (L-dopa and vitamin C) were determined by the 2,2-diphenyl-1-picrylhydrazyl (DPPH) free radical scavenging assay and the 2,2′-azino-bis (3-ethylbenzothiazoline-6-sulfonic acid (ABTS) free radical scavenging assays as Lim et al. [26] described with some modifications. The radical scavenging ability of samples were expressed as mg of vitamin C equivalent (VCE)/gram d.w. The standard compound antioxidant activities were expressed by DPPH/ABTS radical scavenging ability (%) [27].

For DPPH activity, 30 μL sample (2 mg/mL of leaf; 50 mg/mL of seed) or standard compound solution was mixed with 970 μL DPPH solution. After 30 min’s dark incubation, the absorbance was measured at 517 nm. The DPPH solution was prepared with the concentration of 0.1 mM by 80% methanol (*v*/*v*), and the absorbance value was adjusted to 0.70 ± 0.05 at 517 nm. 

For ABTS activity, 10 mM ABTS which dissolved by DMSO mixed with 8 mM 2,2′-Azobis(2-amidinopropane) dihydrochloride which made by 1X phosohate-suffered saline with the ratio of 1:4. Then, the mixed solution was heated 30 min at 65 ± 5 °C under light condition and shanking frequently. Next, the mixed solution was filtered by a 0.45 μm syringe filter and the absorbance of filtrate was adjusted to 0.70 ± 0.05 at 734 nm. The adjusted solution was mixed with 20 μL sample (2 mg/mL of leaf; 10 mg/mL of seed) or standard compound solution and the absorbance was measured at 734 nm after a 10 min’s incubation at 37 °C.

### 2.9. Statistical Analysis

The samples were processed three times. All results were expressed as the mean ± standard error of triplicate experiments. The data were analyzed statistically using SAS software (Enterprise Guide 7.1 version; SAS Institute Inc., Cary, NC, USA). Significant differences between experimental groups were evaluated using ANOVA followed by Tukey’s honestly significant difference (HSD) test at a level of *p* < 0.05. Correlation analysis was carried out by calculating Pearson’s correlation coefficients.

## 3. Results and Discussion

### 3.1. Color Parameters of Young and Old Faba Leaves, and Faba Seeds after Thermal Treatment

**Figure 1:**
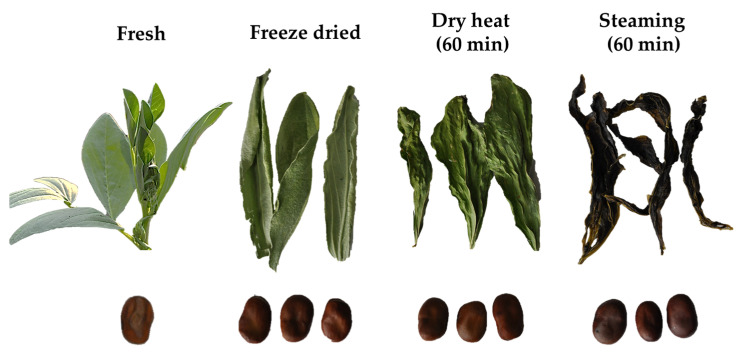
Morphological changes in faba leaves and seeds before and after heat treatment.

**Figure 2:**
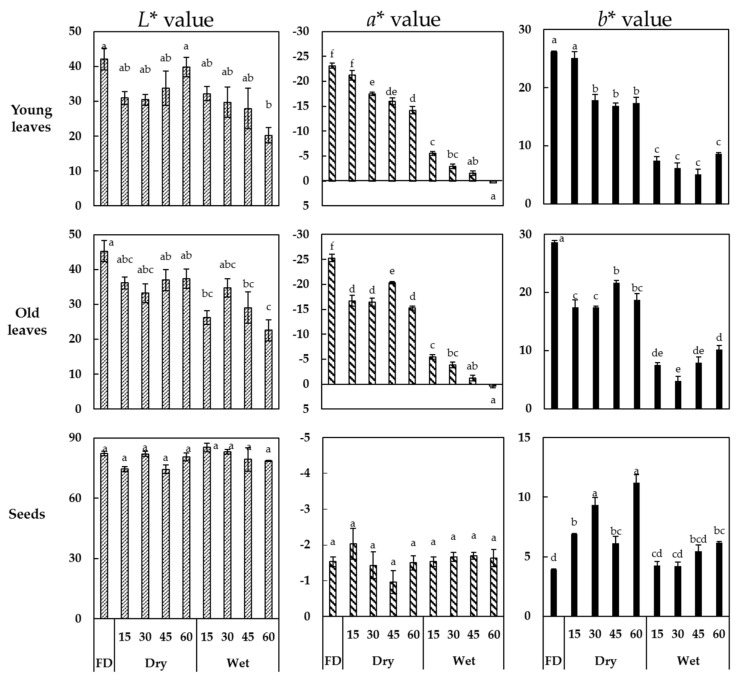
Color parameters (CIE L*, a*, b*) of young and old faba leaves and faba seeds after thermal treatment. FD indicates freeze-dried samples. Dry and wet indicates dry heat and steaming treatments, respectively, with numbers indicating treatment times of 15, 30, 45 and 60 min. Different letters indicate a significant difference between mean values of color parameters at *p* < 0.05 (Tukey’s HSD test).

The morphology of the faba leaves and seeds before and after thermal treatment is shown in Figure 1. Briefly, after the different treatment methods, significant differences were observed in the morphological characteristics of the leaves compared with those of the seeds. The freeze-dried samples were the most similar to the fresh leaves. The dry heat and steaming treatments mainly affected the leaf shape and size, but steaming also affected the color. We further analyzed the color parameters of the faba leaf and seed powders (Figure 2). For leaves, significant changes in the L*, a*, b* values were also observed in thermally-treated samples compared with those freeze-dried. Dry heat treatment produced no significant variations in the L* value of the treated leaves (young leaves varied from 30.43 to 42.13; old leaves from 33.23 to 45.27), but significant variations in the a* values (young leaves varied from −25.30 to −15.23; old leaves from −23.13 to −14.20) and in the b* values (young leaves varied from 17.43 to 28.67; old leaves from 16.90 to 26.20). This indicated that the redness and blueness of the leaves had increased. These results were similar to those of Zhang [28] who reported on the variations in the color parameters of Japanese angelica (*Angelica keiskei*) leaves after freeze drying, convective drying and vacuum oven drying. As expected, the steaming treatment changed the leaf color more strongly than dry heat, not only increasing the redness and blueness, but also decreasing the lightness. This could be explained by the rapid destruction of chlorophyll or a non-enzymatic Maillard reaction [29,30]. However, the patterns of color value variation were different for the seeds. There were no significant changes in the L* (varied from 74.33 to 83.00) and a* (varied from −2.03 to −0.97) values. A significant increase in the b* value (varied from 3.93 to 11.23) occurred in all the thermally-treated seeds that may be caused by Maillard reaction.

### 3.2. Changes in L-dopa Content after Thermal Treatment

**Figure 3:**
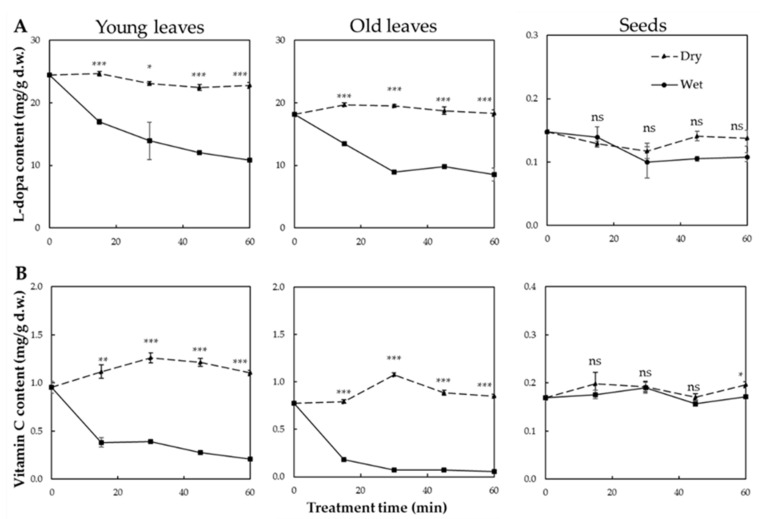
Changes in L-dopa (**A**) and Vitamin C; (**B**) contents after dry and wet heat treatments of young and old faba leaves and faba seeds for different treatment times. Values marked with asterisks are significantly different between the dry and wet heat treatments. (Tukey’s HSD test: * indicates *p* < 0.05, ** indicates *p* < 0.01, *** indicates *p* < 0.001 and ns indicates no significance).

**Figure 4:**
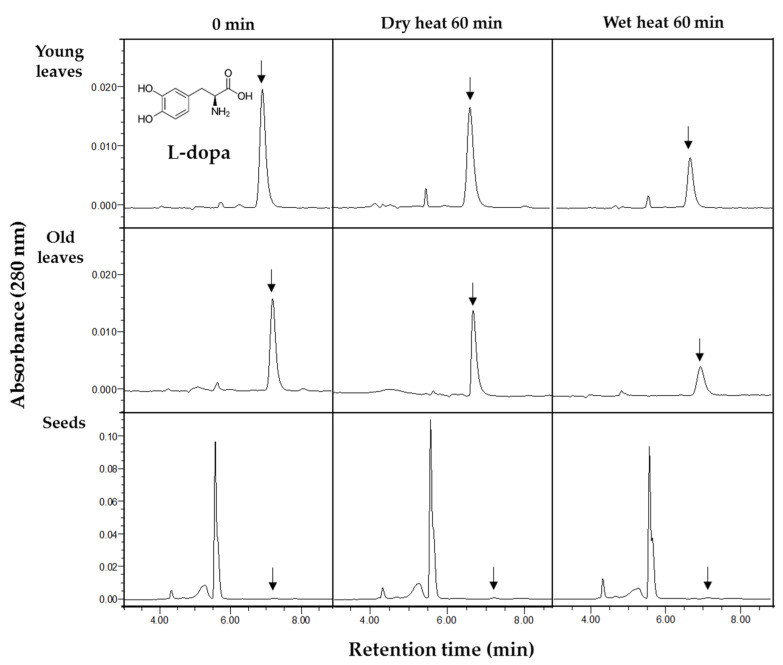
HPLC chromatograms of L-dopa in young and old faba leaves and faba seeds before and after different thermal treatments.

The effect of thermal treatment on L-dopa content in faba leaves and seeds is shown in Figure 3A. Overall, the young leaves contained more L-dopa than the old leaves and seeds. Before thermal treatment (0 min), the young leaves, old leaves and seeds contained 24.44, 18.13 and 0.15 mg/g d.w. of L-dopa, respectively. This agreed with previously reported results where L-dopa had accumulated more in the immature leaves than in the seeds [11]. After processing, similar patterns in the variation of L-dopa content were observed in both the young and old leaves. For the dry heat treatment, there were no significant changes in L-dopa content for treatment times up to 1 h (young leaves ranged from 21.18 to 24.83 mg/g d.w.; old leaves from 17.26 to 20.11 mg/g d.w.). In contrast, for the wet heat treatment, there was a tendency for the L-dopa content to decline rapidly before leveling off as treatment time increased (old leaves decreased from 18.13 to 8.52 mg/g d.w.; young leaves from 24.83 to 10.88 mg/g d.w.). However, the L-dopa content of the seeds was relative stable, ranging from 0.12 to 0.15 mg/g d.w. for dry heat and from 0.10 to 0.15 mg/g d.w. for wet heat. Typical HPLC chromatograms of the variations in L-dopa content of young and old faba leaves, and faba seeds are shown in Figure 4.

L-dopa, an active phenolic compound, has been found in large amounts in faba leaves compared with other faba tissues [11,15]. Thermal treatment usually has a significant effect on the content of phenolic acids as treatment time is prolonged [16]. However, little information is available on the effect of variations in dry and wet thermal treatment times on the L-dopa contents of faba leaves and seeds. Our results show that for treatments up to 1 h, dry heat retained the L-dopa content better than wet heat. Even though there was no significant difference in L-dopa content of the seeds between the dry and wet heat treatments, a relatively higher amount of L-dopa was found in the dry-heated seeds after longer treatment times. This may due the relatively increased extract ratio of L-dopa by dry heat treatment, and the degradation of L-dopa by steaming after long time treatment. Similar results have been reported by Etemadi et al. [31], where the L-dopa contents of faba seeds and leaves were not significantly different between oven drying for 1 day at 50 °C and drying for 7 day at room temperature, whereas boiling for 40 min caused an obvious reduction. This also agreed with Mugendi et al. [32], who reported that roasting at 100 °C did not reduce the L-dopa content of mucuna beans, while autoclaving did. Nyirenda et al. [33] also reported that boiling reduced the L-dopa content of velvet beans (*Mucuna pruriens*). Wet heat is a combination of temperature with high moisture. Thus, it is reasonable to speculate that L-dopa content can easily be reduced under conditions of relative higher moisture and temperatures. The relative stability of the L-dopa content in the faba seeds can be explained by the hard seed structure and the relatively lower internal water content reducing the transfer of short-time thermal energy [34]. This assumption is also supported by Pappert et al. [35], who demonstrated the instability of L-dopa solution which degraded naturally over time, and by Zhou et al. [36], who showed that L-dopa standard solution oxidized under alkaline conditions and became unstable under thermal stress.

### 3.3. Changes in Vc Content after Thermal Treatment

Vitamin C has been widely used as a traditional antioxidant in the daily diet and also in commercial applications. Edible green vegetables are usually good sources of Vc. Many studies have determined the Vc content in various green vegetables such as spinach, broccoli and cabbage [37]. De Cillis et al. [38] have also reported the Vc content (386–703 mg/100 g fresh weight) in 6 genotypes of immature faba beans. As faba leaves are considered an unconventional vegetable, their Vc content has not yet been reported so the Vc content of faba leaves at two levels of maturity were compared with that of mature faba seeds and its variation with different thermal treatments was determined. Figure 3B shows that freeze-dried young and old faba leaves contained 0.95 and 0.77 mg/g d.w. of vitamin C, respectively, similar values to those reported by Zhang et al. [39] in bitter melon green leaves (ranged from 0.85 to 1.22 mg/g d.w.) and by Shofian et al. [40] in freeze-dried mango fruits (8.34 mg/100 g of fresh weight which moisture content around 88.67%). The mature faba seeds only contained 0.17 mg/g d.w. of Vc, which was a quarter that of the old leaves. However, our results were higher than those of Moriyama et al. [41], who reported a Vc content of 0.21–2.01 mg/100 g d.w. in three genotypes of dehydrated faba seeds and also in other legume seeds such as yard bean, kidney bean and soybean. The higher content of Vc in our results may be the results of different phenotypes or extraction methods.

After thermal treatment, different patterns of variation in Vc content were observed between the leaves and the seeds. For leaves, dry heat slightly increased the Vc content, reaching a maximum of 1.26 and 1.08 mg/g d.w. for young and old leaves, respectively, after a 30-min treatment. In contrast, steaming caused a significant decrease in Vc content of up to 90% as the treatment time increased. This result was similar to that of Lee et al. [37], who reported that steaming significantly reduced the Vc content in several vegetables such as chard, potato, sweet potato and spinach, whereas microwave treatment increased the Vc content. Vc is a water soluble and heat-sensitive compound, whose degradation depends on temperature and moisture [42] which may explain the significant reduction in the Vc content of steamed leaves [19]. For the faba seeds, the Vc content was relatively stable compared with the leaves after thermal treatment. A slight increase in Vc content (0.20 mg/g d.w.) was observed after 60 min of dry heat treatment but there was no significant change in Vc content after 60 min of steaming. This may have been the effect of a lower internal water content and water activity of the food matrix, which reduced the degradation of Vc [19]. The pattern of variation in Vc content after thermal treatment was different compared with that of L-dopa content (Figure 3) because of the differences in compound structure and thermal sensitivity. 

### 3.4. Total Phenolics Content

The changes in the total phenolics content (TPC) of the faba leaves and seeds after thermal treatment are shown in Table 1. For the non-thermally treated group (0 min), the TPC of the young leaves (54.31 mg GAE/g d.w.) was 1.25 times higher than that of the old leaves. This result agreed with previous reports that a higher TPC was found in the immature than in the mature leaves of *Melia azedarach* L. (Chinaberry) [43] and *Coffea arabica* L. (Coffee) [44]. This could be explained by the higher enzyme activity and gene expression level related to the synthesis of phenolic compounds in immature leaves [43]. However, compared with faba leaves, there was small amounts of TPC (3.86 mg GAE/g d.w.) found in faba seeds. Similar results have also been reported for adzuki beans whose leaves were a more valuable source of polyphenolic substances than the seeds [45]. Several studies have quantified TPC in faba tissues. Lu et al. [6] evaluated the distribution of TPC in seed coats (ranged from 1.62 to 4.71 mg GAE/g d.w.) and cotyledons (ranged from 0.03 to 0.27 mg GAE/g d.w.) of five immature faba bean varieties. Randhir et al. [46] reported the increase of TPC (around 1.1 to 2.5 mg GAE/g fresh weight) in faba bean sprouts in 8 days. Chaieb et al. [47] investigated the TPC in fourteen faba genotypes of pods (ranged from 56.97 to 149.21 mg GAE/g d.w.) and whole plants (ranged from 92.85 to 157.68 mg GAE/g d.w.). However, the present study is the first to report on the distribution of TPC in faba leaves of different maturity and faba seeds. Here, it is important to point out the limitations of Folin-Ciocalteu method what we used in determination of TPC. Expect phenolic compounds, the presence of interfering substances (Vc, dehydroascorbic acid, reducing sugars, etc) in plant extracts can also react with Folin-Ciocalteu reagent, which lead to impact on hampering the accuracy of the assay [48,49]. Beside this, the different extraction methods (solvent, time, temperature, etc.) may also affect to the TPC in plants.

After wet heat treatment, the TPC of old leaves decreased from 43.18 to 21.59 mg GAE/g d.w. and of young leaves from 54.31 to 24.21 mg GAE/g d.w., but there were no significant changes in TPC after dry heat treatment for up to 1 h where the TPC of old leaves ranged from 43.18 to 47.29 mg/g d.w. and of young leaves from 52.87 to 55.60 mg/g d.w. These results agreed with those of Hwang et al. [50] on peppers, who reported that dry heat (stir-frying and roasting) was better for maintaining TPC than wet heat (boiling and steaming) after treatments of 5, 10 and 15 min and were also similar to those of Gunathilake et al. [51], who suggested that wet heat (boiling and steaming) significantly decreased the TPC in leaves of *Olax Zeylanica* (mella), *Sesbania grandiflora* (kathurumurunga) and *Passiflora edulis* (passion fruit). However, the thermal processing did not significantly affect the TPC of faba seeds which ranged from 3.67 to 4.31 mg/g d.w. Siah et al. [52] reported a decrease in the phenolics content of faba beans after soaking, boiling and autoclaving. A significant reduction in the TPC of faba seeds has also been reported after a roasting treatment [53]. This may have been caused by the processing methods and by differences in the thermal treatment time. Phenolic compounds are usually responsible for the antioxidant activity of a food matrix, with higher phenolics contents usually indicating a higher antioxidant activity. However, although faba leaves are usually eaten as a vegetable after thermal treatment, there have been no reports on their phenolics content at different levels of maturity or changes caused by thermal treatment. The results suggest that consuming leaves rather than seeds would be a good method of acquiring phenolic compounds and that young faba leaves, either raw, treated by dry heat or by wet heat for a short time, should be eaten to increase the intake of phenolic compounds. 

### 3.5. Changes in Total Flavonoids Content

Changes of total flavonoids content (TFC) of faba leaves and seeds after thermal processing are shown in Table 1. Briefly, the pattern in the variation of TFC after thermal treatment was similar to that observed for TPC. For the freeze-dried faba leaves, the TFC of young leaves (66.96 mg CE/g d.w.) and old leaves (43.68 mg CE/g d.w.) was at least 43 times greater than that of faba seeds (1.00 mg CE/g d.w.). After thermal treatment, different patterns of variation in TFC were observed between the leaves and the seeds. For the leaves, short-time steaming (up to 30 min) caused a significant reduction (around 50%) in the TFC, then tended to stabilize as treatment time increased. For seeds, neither type of thermal treatment significantly affected the TFC. 

Flavonoids, widely distributed in plants, play an important role in propagation, resisting insects, disease prevention and defending against environmental changes [54] so may explain why a higher TFC was observed in the leaves than in the seeds. Because of the health benefits of flavonoids in the human diet, characterizing flavonoids in faba seeds and leaves has been widely studied. Jin et al. [55] reported that faba bean seeds are abundant in proanthocyanidins especially procyanidin- and prodelphinidin-type flavan-3-ol subunits. Neugart et al. [10] investagted the main flavonol glycosides in four different cultivars of faba leaves were kaempferal with primarily glucose, rhamnose, and galactose as sugar moieties. However, the distribution of flavonoids in faba leaves of different maturity and the effects of thermal processing on TFC have been first reported in the present study. The results, showing that steaming significantly reduced the TFC of faba leaves, are consistent with those of Gunathilake et al. [51], who reported that steaming caused a reduction in the TFC of *Passiflora edulis* leaves compared with the raw leaves, and with those of Agbo et al. [56], who suggested that the TFC of sweet potato leave steamed for 20 min decreased significantly compared with that of the fresh leaves. Flavonoids are relatively heat-sensitive polyphenolic compounds [17] which suggests that high humidity is an important factor in reducing flavonoids in faba leaves. As aforementioned that steaming is a combination of temperature with high moisture, which accelerates the transfer of energy compared with dry heat and thus causes the chemical conversion or destruction of the flavonoid structure. The results showed that there were no significant changes in TFC between steaming treatments of 30, 45 and 60 min which can be explained by the degradation of heat-sensitive flavonoid compounds, with the relatively heat-stable substances remaining. Therefore, the different patterns in TFC variation between faba leaves and seeds may have been caused by differences in the flavonoid composition and properties of the materials. 

### 3.6. DPPH and ABTS Radical Scavenging Abilities

The effect of thermal treatment on the antioxidant activity of the faba leaves and seeds was evaluated by their DPPH and ABTS radical scavenging abilities. The young leaves exhibited a higher antioxidant activity than the old leaves and seeds whether thermally treated or not. The initial antioxidant activities of freeze-dried young leaves (73.70 mg VCE/g d.w. for DPPH; 52.96 mg VCE/g d.w. for ABTS), old leaves (54.55 mg VCE/g d.w. for DPPH; 37.70 mg VCE/g d.w. for ABTS) and seeds (1.47 mg VCE/g d.w. for DPPH; 6.61 mg VCE/g d.w. for ABTS) are shown in Table 1. After dry heat treatment, there were no significant changes in the DPPH and ABTS radical scavenging abilities in leaves. In contrast, steaming caused a significant reduction in the DPPH (old leaves decreased from 54.55 to 27.48 mg VCE/g d.w.; young leaves from 73.70 to 40.54 mg VCE/g d.w.) and the ABTS (old leaves decreased from 37.70 to 17.00 mg VCE/g d.w.; young leaves from 52.96 to 22.95 mg VCE/g d.w.) radical scavenging abilities. Unlike the leaves, the seeds exhibited an increase in ABTS radical scavenging ability (from 6.61 to 7.55 mg VCE/g d.w.) after a long dry heat treatment, but this did not occur after steaming. This may have been caused by the accelerated extraction rate after the dry heat treatment. However, there were no significant changes in DPPH radical scavenging ability after thermal treatment.

Previous studies evaluating the antioxidant activities of faba tissues have mainly focused on different genotypes of seeds, seed coats and cotyledons, sprouts and pods [6,46,57]. The antioxidant activity of faba leaves has been verified as higher than several commercial vegetables such as asparagus, broccoli raab, spinach and cabbage [4]. However, to the best of our knowledge, this is the first study to compare the effect of thermal treatment on the antioxidant activity of faba seeds and faba leaves at different levels of maturity. The present study has shown that faba leaves, especially young leaves, possess higher antioxidant activities than seeds after same treatments, possibly because of the higher content of phytochemicals such as phenolic acids and flavonoids, which are usually highly positively related to antioxidant activity [28]. In the case of seeds, many antioxidant components, like anti-photooxidative compounds shown to leaves relatively less contained and exhibited weak correlation with antioxidant activity. This can also be explained by the different compositions of phytochemicals which exhibit different antioxidant potential [10,55,58]. This could also explain why faba leaves showed a strong antioxidant activity based on DPPH radical scavenging ability, whereas faba seeds showed a strong antioxidant activity based on ABTS radical scavenging ability.

### 3.7. Correlation Analysis and the Antioxidant Activity of Pure Standard Compounds

To illustrate the potential contribution to antioxidant activity of the active compounds in the faba leaves and seeds, Pearson’s correlation coefficients were determined on the relationships between DPPH/ABTS radical scavenging ability and the contents of the metabolite groups (Table 2). For both young and old faba leaves, the DPPH and ABTS radical scavenging abilities were significantly correlated (*p* < 0.001) with the L-dopa and Vc contents, TPC and TFC. Similarly, a good correlation between antioxidant activity and L-dopa content was also observed in the faba seeds. These results were similar to those of Okumura et al. [57], who reported that L-dopa contributed to the antioxidant activity of faba bean sprouts. However, there were no significant correlations between Vc content, TPC, TFC and antioxidant activity for faba seeds. This could be explained by the antioxidant activity of faba seeds mainly originating from the seed coat rather than the cotyledon [6], with the seed coat only forming a small proportion of the whole seed.

**Figure 5:**
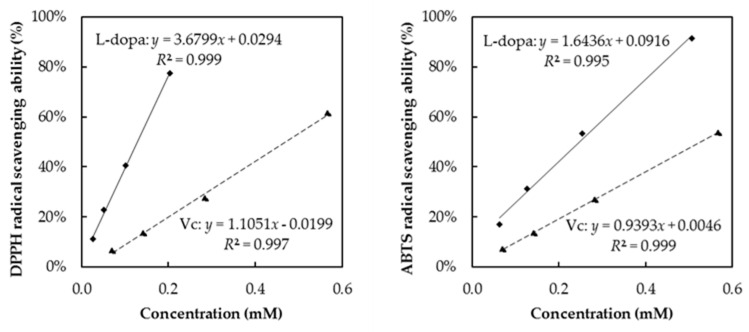
Antioxidant activity of standard compounds of L-dopa and vitamin C.

Vc, phenolics and flavonoids can be considered as the main phytochemicals in plants (food matrix) that contribute to antioxidant activity. In the present study, L-dopa, a phenolic acid, which accumulates in Fabaceae, has also been found to be an important contributor to the antioxidant activity of faba leaves and seeds. Therefore, the pure standard compound antioxidant of L-dopa was measured and compared with that of Vc (Figure 5). The strong DPPH/ABTS radical scavenging abilities of the L-dopa standard were verified as being stronger than those of Vc. Similar results, that L-dopa is an effective antioxidant, have also been reported by Gülçin [59] using different in vitro assays such as DPPH/ABTS radical scavenging ability, reductive ability and were compared with the synthetic antioxidants, BHA and BHT.

## 4. Conclusions

This study evaluated the color, L-dopa and Vc contents, TPC and TFC and antioxidant activity of fully mature faba seeds and of leaves at two levels of maturity after a 1-h thermal treatment. Overall, faba leaves, especially at a young stage, are good sources of L-dopa and Vc, with high TPC and TFC, thus possessing a stronger antioxidant activity than the seeds. In detail, for the leaves, the dry heat treatment significantly influenced the color parameters (increasing redness and blueness) rather than the nutritional values and bioactivities, whereas steaming significantly reduced several of these parameters. For seeds, compared with freeze dried, there were no significant changes in the color parameters except for a slight increase in yellowness, with no significant changes in nutritional values except for an increase in ABTS radical scavenging ability after dry heat treatment. Correlation analysis found a positive correlation between L-dopa content and radical scavenging ability in both the leaves and seeds. The stronger radical scavenging of the L-dopa standard compound compared with vitamin C was verified. In conclusion, faba leaves and seeds are valuable sources for consuming phytochemicals. Faba leaves should be eaten rather than faba seeds, either raw, treated by dry heat or steamed for a short time. L-dopa is a major contributor to the antioxidant activity of faba leaves and seeds. Finally, we can also propose that faba leaves offer a high potential for use in the food and pharmaceutical industries, not only because of their beneficial effect on Parkinson’s Disease, but also because of their strong antioxidant activity.

## Figures and Tables

**Table 1:** Changes in TPC, TFC and antioxidant activities (DPPH and ABTS radical scavenging ability) after thermal treatment of young and old faba leaves and faba seeds.

		Freeze Dried (0 Min)	Dry Heat				Wet Heat			
			15 Min	30 Min	45 Min	60 Min	15 Min	30 Min	45 Min	60 Min
TPC (mg GAE/g d.w.)	Young leaves	54.31 ± 0.72 ^a^	55.60 ±2.11 ^a^	52.87 ±1.03 ^a^	52.91 ± 1.72 ^a^	53.09 ± 3.02 ^a^	35.24 ± 1.14 ^b^	30.96 ± 6.35 ^b^	26.30 ± 0.27 ^b^	24.21 ± 0.53 ^b^
	Old leaves	43.18 ± 0.50 ^a^	44.92 ± 1.00 ^a^	46.78 ± 0.74 ^a^	46.20 ± 1.29 ^a^	47.29 ± 1.79 ^a^	31.29 ± 0.60 ^b^	23.28 ± 0.83 ^c^	25.58 ± 0.31 ^c^	21.59 ± 0.35 ^c^
	Seeds	3.86 ± 0.11 ^a^	4.13 ± 0.15 ^a^	4.08 ± 0.23 ^a^	4.31 ± 0.22 ^a^	4.11 ± 0.21 ^a^	3.76 ± 0.20 ^a^	3.67 ± 0.10 ^a^	3.67 ± 0.21 ^a^	3.74 ± 0.28 ^a^
TFC (mg CE/g d.w.)	Young leaves	63.67 ± 1.26 ^a^	58.87 ± 2.97 ^a^	57.92 ± 1.27 ^a^	53.22 ± 1.63 ^ab^	56.64 ± 1.71 ^a^	37.84 ± 0.09 ^bc^	30.80 ± 8.19 ^c^	28.85 ± 0.35 ^c^	29.99 ± 1.09 ^c^
	Old leaves	43.68 ± 0.22 ^a^	43.76 ± 1.55 ^a^	45.98 ± 1.90 ^a^	44.12 ± 0.53 ^a^	43.64 ± 2.13 ^a^	29.58 ± 0.74 ^b^	20.36 ± 1.43 ^c^	22.48 ± 0.53 ^c^	23.12 ± 1.73 ^c^
	Seeds	1.00 ± 0.06 ^a^	1.02 ± 0.03 ^a^	0.94 ± 0.05 ^a^	1.06 ± 0.01 ^a^	0.97 ± 0.06 ^a^	1.20 ± 0.06 ^a^	1.05 ± 0.11 ^a^	1.18 ± 0.09 ^a^	1.12 ± 0.24 ^a^
DPPH (mg VCE/g d.w.)	Young leaves	73.70 ± 0.47 ^a^	71.81 ± 0.84 ^a^	70.89 ± 0.87 ^a^	68.40 ± 1.39 ^a^	69.62 ± 1.22 ^a^	51.94 ± 0.96 ^b^	43.31 ± 9.40 ^b^	41.71 ± 0.57 ^b^	40.54 ± 0.67 ^b^
	Old leaves	54.55 ± 0.72 ^a^	56.96 ± 0.57 ^a^	58.87 ± 0.59 ^a^	58.67 ± 0.88 ^a^	56.86 ± 1.39 ^a^	41.19 ± 0.52 ^b^	27.48 ± 1.37 ^d^	33.12 ± 0.44 ^c^	30.21 ± 1.96 ^cd^
	Seeds	1.47 ± 0.06 ^a^	1.29 ± 0.27 ^a^	1.15 ± 0.13 ^a^	1.49 ± 0.08 ^a^	1.30 ± 0.07 ^a^	1.19 ± 0.04 ^a^	1.15 ± 0.12 ^a^	1.41 ± 0.08 ^a^	1.36 ± 0.25 ^a^
ABTS (mg VCE/g d.w.)	Young leaves	52.96 ± 3.92 ^a^	54.67 ± 0.76 ^a^	50.74 ± 1.18 ^a^	52.57 ± 1.48 ^a^	53.10 ± 0.29 ^a^	30.03 ± 3.93 ^b^	26.87 ± 0.96 ^b^	26.10 ± 2.29 ^b^	22.95 ± 2.67 ^b^
	Old leaves	37.70 ± 1.46 ^a^	39.47 ± 2.20 ^a^	42.71 ± 0.65 ^a^	43.20 ± 1.46 ^a^	42.55 ± 0.89 ^a^	19.25 ± 2.49 ^b^	15.76 ± 1.58 ^b^	19.13 ± 1.80 ^b^	17.00 ± 1.95 ^b^
	Seeds	6.61 ± 0.09 ^bc^	6.97 ± 0.08 abc	6.64 ± 0.22 ^bc^	7.55 ± 0.24 ^a^	7.17 ± 0.16 ^ab^	6.27 ± 0.13 ^c^	6.39 ± 0.12 ^bc^	6.32 ± 0.19 ^c^	6.31 ± 0.14 ^c^

Values are averages with standard errors from triplicate experiments. Different letters within same line indicate significant differences at *p* < 0.05 (Tukey’s HSD test).

**Table 2:** Correlation coefficients between antioxidant activities and metabolite groups assembled with processed faba samples.

		L-dopa	Vitamin C	TPC	TFC	DPPH	ABTS
Young leaf	L-dopa	1					
	vitamin C	0.890 ***	1				
	TPC	0.977 ***	0.918 ***	1			
	TFC	0.861 ***	0.888 ***	0.882 ***	1		
	DPPH	0.826 ***	0.891 ***	0.833 ***	0.948 ***	1	
	ABTS	0.907 ***	0.923 ***	0.919 ***	0.910 ***	0.919 ***	1
Old leaf	L-dopa	1					
	vitamin C	0.954 ***	1				
	TPC	0.971 ***	0.969 ***	1			
	TFC	0.960 ***	0.963 ***	0.980 ***	1		
	DPPH	0.984 ***	0.967 ***	0.985 ***	0.973 ***	1	
	ABTS	0.933 ***	0.967 ***	0.960 ***	0.941 ***	0.956 ***	1
Seeds	L-dopa	1					
	vitamin C	0.229 ^ns^	1				
	TPC	0.256 ^ns^	0.103 ^ns^	1			
	TFC	0.079 ^ns^	−0.340 ^ns^	0.043 ^ns^	1		
	DPPH	0.495 **	0.079 ^ns^	0.179 ^ns^	0.309 ^ns^	1	
	ABTS	0.566 **	0.228 ^ns^	0.480 *	−0.252 ^ns^	0.396 *	1

Tukey’s HSD test, * indicates *p* < 0.05, ** indicates *p* < 0.01, *** indicates *p* < 0.001, and ns indicates no significance. Data of L-dopa, vitamin C, TPC, TFC, DPPH, and ABTS for correlation analysis were calculated by the time-dependent dry and wet heat values, ranging 0 to 60 min treatments. The information of sample concentrations was mentioned in method sections.

## Data Availability

Data is contained within the article.
